# The potential effects of deploying SARS-Cov-2 vaccines on cold storage capacity and immunization workload in countries of the WHO African Region

**DOI:** 10.1016/j.vaccine.2021.02.037

**Published:** 2021-04-08

**Authors:** Justin R. Ortiz, Joanie Robertson, Jui-Shan Hsu, Stephen L. Yu, Amanda J. Driscoll, Sarah R. Williams, Wilbur H. Chen, Meagan C. Fitzpatrick, Samba Sow, Robin J. Biellik, Kathleen M. Neuzil

**Affiliations:** a Center for Vaccine Development and Global Health, 685 W. Baltimore St., University of Maryland School of Medicine, Baltimore, MD, USA; b PATH, 2201 Westlake Avenue, Suite 200, Seattle, WA, USA; c Division of Pulmonary and Critical Care Medicine, 110 S. Paca St, University of Maryland School of Medicine, Baltimore, MD, USA; d Centre pour le Développement des Vaccins, Ministère de la Santé, BP251Bamako, Mali; e Independent Consultant, Tranchepied 10, 1278 La Rippe, Switzerland

**Keywords:** Immunization, Africa, SARS-CoV-2, Implementation, Delivery

## Abstract

**Background:**

SARS-CoV-2 vaccines will be deployed to countries with limited immunization systems.

**Methods:**

We assessed the effect of deploying SARS-Cov-2 vaccines on cold storage capacity and immunization workload in a simulated WHO African Region country using region-specific data on immunization, population, healthcare workers (HCWs), cold storage capacity (quartile values for national and subnational levels), and characteristics of an approved SARS-CoV-2 vaccine. We calculated monthly increases in vaccine doses, doses per vaccinator, and cold storage volumes for four-month SARS-CoV-2 vaccination campaigns targeting risk groups compared to routine immunization baselines.

**Results:**

Administering SARS-CoV-2 vaccines to risk groups would increase total monthly doses by 27.0% for ≥ 65 years, 91.7% for chronic diseases patients, and 1.1% for HCWs. Assuming median nurse density estimates adjusted for absenteeism and proportion providing immunization services, SARS-CoV-2 vaccination campaigns would increase total monthly doses per vaccinator by 29.3% for ≥ 65 years, 99.6% for chronic diseases patients, and 1.2% for HCWs. When we applied quartiles of actual African Region country vaccine storage capacity, routine immunization vaccine volumes exceeded national-level storage capacity for at least 75% of countries, but subnational levels had sufficient storage capacity for SARS-CoV-2 vaccines for at least 75% of countries.

**Conclusions:**

In the WHO African Region, SARS-CoV-2 vaccination campaigns would substantially increase doses per vaccinator and cold storage capacity requirements over routine immunization baselines. Pandemic vaccination campaigns would increase storage requirements of national-level stores already at their limits, but sufficient capacity exists at subnational levels. Immediate attention to strengthening immunization systems is essential to support pandemic responses.

## 1 Background

There is a robust pipeline of SARS-CoV-2 vaccine candidates in various stages of development [1], [2]. While the challenges of developing a vaccine at an accelerated timeline for this newly-emerged pathogen have been well described [2], less attention has been given to the challenges of vaccine deployment and delivery. During the 2009 influenza A (H1N1) pandemic, vaccine deployment to many developing countries was suboptimal. WHO reported deploying 32,096,290 doses of 2009 H1N1 vaccine to WHO African Region (subsequently called “African Region”) countries, of which 62% were utilized [3]. African Region countries responding to a post pandemic survey reported 16.7% of target populations were vaccinated, with total population coverage of 1% [4]. Most of these vaccine doses arrived after June 2010, despite the fact that H1N1 vaccines were prequalified by WHO for procurement beginning November 2009 [3]. This delay, largely due to obstacles in vaccine deployment and delivery [3], resulted in many preventable illnesses.

The SARS-CoV-2 pandemic has already caused more severe health impact than the 2009 H1N1 pandemic [5], and national governments, and technical and partner agencies aspire to higher population vaccine coverage than what was achieved during the prior pandemic [4], [6], [7]. A WHO report following the 2009 H1N1 pandemic concluded that existing health systems, immunization infrastructures, and deployment training played key roles during the vaccine response [8]. Critical to immunization infrastructure is the cold chain, defined as the series of actions and equipment necessary to maintain a vaccine within a specific low temperature range from production to the point of administration. While the number of deployed H1N1 vaccine doses covered only a fraction of at-risk populations identified, 29% of countries of the African Region reported having insufficient cold chain and logistics capacity for a comprehensive pandemic response [4].

Since 2014, a concerted global effort has improved cold chain infrastructure in developing countries [9], [10]. A recent self-assessment of SARS-CoV-2 vaccine program readiness by African Region countries, however, indicated only 44% have coordination structures in place, and fewer have adequate resources, data collection and monitoring tools, and communication plans necessary for pandemic vaccine response [11]. We undertook this study to investigate the impact SARS-CoV-2 vaccination programs would have on vaccine delivery and storage systems in the African Region. Our goal was to assess critical operational aspects of mass vaccination programs targeting SARS-CoV-2 risk groups.

## 2 Methods

We used tools and methods standard to cold chain capacity assessments that are performed routinely by countries in preparation for new vaccine introductions [12]. We aimed to explore aspects of African Region-level SARS-CoV-2 vaccine implementation. This analysis is not intended to take the place of ongoing, country-level assessments using national contexts and data for deployment planning [13]. Rather than describe any individual country or countries, we simulated a 20 million population country using the 2017 age distribution for the African Region [14]. For this manuscript, we refer to standard, non-pandemic immunization programs as “routine” immunization and SARS-CoV-2 immunization programs as mass-vaccination “campaigns.” We compared the number of doses and vaccine storage volumes required for routine immunization alone and with SARS-CoV-2 mass vaccination campaigns. We then compared the required vaccine storage volumes for both scenarios to actual vaccine cold storage capacities from countries in the African Region. Our outcomes of interest included the monthly percentage increases in vaccine doses to be administered, doses administered per vaccinator, and cold storage volume requirements for SARS-CoV-2 mass vaccination campaigns compared to routine immunization baselines.

### 2.1 Immunization strategies

We used a standard routine immunization schedule for the simulated country based on current WHO policy recommendations (Table 1) [15]. We then adopted a SARS-CoV-2 vaccination campaign strategy targeting the following risk groups identified by WHO: persons aged ≥ 65 years, persons with certain chronic medical conditions (chronic diseases), healthcare workers (HCWs), and all risk groups combined [7]. WHO defines the HCW risk group as “doctors, nurses and midwives and community healthcare workers [7].” We assumed the SARS-CoV-2 vaccination campaign would span four months in duration and would require two doses, one month apart. To determine the fraction of the population in the chronic diseases group, we applied WHO-recommended [16], age-based estimates of comorbid risk factors for severe SARS-CoV-2 illness specific to the African Region [17]. Comorbidities included chronic cardiovascular, respiratory, neurologic, kidney and/or liver diseases; diabetes; cancers; HIV/AIDS; tuberculosis; and sickle cell disorders [17]. For HCWs, we used WHO estimates of skilled health personnel for the African Region, distributing these workers evenly across ages 20 through 64 years [18]. For the all risk groups combined category, we assumed HCWs had the same prevalence of chronic diseases as the general population and adjusted to avoid double counting.

**Table 1:** Routine and SARS-CoV-2 vaccines, schedules, and cold storage volume per dose.

	Schedule	Cold storage tertiary packaging volume per dose (mL)	Cold storage secondary packaging volume per dose (mL)	Presentation	Product used
Routine infant immunization					
Bacille Calmette-Guerin	Birth dose	4.98	1.44	10 dose MDV	Japan BCG Laboratory
Hepatitis B	Birth dose	12.56	2.86	10 dose MDV	Euvax B (paed)
Diphtheria-tetanus-pertussis-hepatitis B-Haemophilus influenzae type b (pentavalent)	6, 10, and 14 weeks	16.70	3.06	10 dose MDV	Eupenta
Polio (oral)	6, 10, and 14 weeks	6.22	1.40	10 dose MDV	PT Bio Farma (Persero)
Polio (inactivated)	6 weeks	23.95	4.00	5 dose MDV	Bilthoven Biologicals
Pneumococcal (conjugate)	6, 10, and 14 weeks	36.28	3.60	4 dose MDV	Pfizer
Rotavirus	6 and 10 weeks	49.45	46.30	1 dose SDV	Merck
Measles-rubella	9–12 months, 13–24 months	9.84	2.11	10 dose MDV	Serum Institute of India Pvt. Ltd.
Tetanus-diphtheria	13–24 months	9.47	2.38	10 dose MDV	PT Bio Farma (Persero)
Meningococcal A (conjugate)	13–24 months	9.84	2.11	10 dose MDV	Serum Institute of India Pvt. Ltd.
Yellow fever	13–24 months	3.59	2.99	10 dose MDV	Bio-Manguinhos/Fiocruz
Routine child immunization					
HPV (girls only)	2 doses from 9 to 14 years	7.61	4.84	2 dose MDV	GlaxoSmithKline Biologicals SA
Tetanus-diphtheria	9–14 years	9.47	2.38	10 dose MDV	PT Bio Farma (Persero)
SARS-CoV-2 immunization targeting risk groups					
≥65 years	2 doses, 1 month apart	2.61	2.10	10 dose MDV	AstraZeneca/Oxford University SARS-CoV-2 vaccine characteristics produced by Serum Institute of India
Healthcare workers	2 doses, 1 month apart	2.61	2.10	10 dose MDV	AstraZeneca/Oxford University SARS-CoV-2 vaccine characteristics produced by Serum Institute of India
Chronic diseases	2 doses, 1 month apart	2.61	2.10	10 dose MDV	AstraZeneca/Oxford University SARS-CoV-2 vaccine characteristics produced by Serum Institute of India

Notes: 1. Vaccines and schedules are from WHO Immunization Tables except SARS-CoV-2 vaccines [38]. SARS-CoV-2 vaccines use the dose schedule for AstraZeneca/Oxford University SARS-CoV-2 vaccine characteristics produced by Serum Institute of India, which is similar to other licensed SARS-CoV-2 vaccines in Europe and the United States. 2. Tertiary and secondary packaging volumes per dose from WHO Prequalified Vaccines Database with the exception of SARS-CoV-2 vaccines [19]. 3. SARS-CoV-2 cold storage volume per dose use AstraZeneca/Oxford University SARS-CoV-2 vaccine characteristics produced by Serum Institute of India. 4. HPV vaccination programs generally target girls only aged 9 through 14 years [38]. For this simulation, we assumed the full HPV immunization series was given to girls 9 years of age.

### 2.2 Vaccine storage

We identified WHO prequalified routine vaccine products [19], and we recorded the cold storage volumes required per dose for the standard routine immunization schedule (Table 1). We prioritized ten-dose vials for the schedule whenever they were available, choosing the product with the median secondary packaging volume if there were multiple prequalified ten-dose vial products. For SARS-CoV-2 vaccines, we used 10 dose vials with cold storage volume per dose required for the SARS-CoV-2 vaccine developed by AstraZeneca/Oxford University and produced by Serum Institute of India. Cold storage volume is the total volume of vaccines maintained at refrigerated temperatures throughout storage and transport. Cold storage in countries is organized by levels, with the national level being where procured vaccines are received and stored before distribution to subnational levels (region, district, and health facility). Vaccines (and diluents, for reconstituted products) are produced by the manufacturer in vials or other primary packaging that are then packed together in labeled boxes called “secondary packaging” [19]. Products in secondary packaging are packed in cartons called “tertiary packaging” [19]. Vaccines are typically maintained in tertiary packaging at the national level [20]. Tertiary packaging volumes per dose are often around ten times the secondary packaging volumes per dose due to additional insulation and thermal packaging materials. At subnational levels, packaging materials are removed and vaccines are stored in their secondary packaging [20], [21]. Cold storage temperature for routine vaccines is typically 2° to 8 °C, although some more thermostable products are becoming available [19]. Vaccine vials used in most developing countries carry individual thermo-chemical temperature monitors that indicate when a vial has been outside of the recommended storage temperature range and should be discarded [22], [23].

### 2.3 Vaccine storage and distribution

We estimated vaccine storage volumes using WHO tools and guidance [12], [19], [21], [22], [24]. We compared routine immunization to SARS-CoV-2 vaccination campaigns according to evidence-based assumptions about vaccine wastage (doses that are damaged or unused) [25], reserve stock (excess supply in case of increased demand or stock outs) [12], [22], [26], and resupply intervals (Table 2) [24]. In the case of SARS-CoV-2 vaccines, we assumed 3% wastage, 0% reserve stock, and monthly resupply intervals given anticipated high global demand and limited supply. We received summary vaccine cold storage capacity data from Gavi, the Vaccine Alliance, for African Region countries that are eligible to receive Gavi support. These data included the minimum, 25th percentile, 50th percentile, 75th percentile, and upper range values of total country storage capacities, as well as the median proportion of national-level stores and health facility-level stores to total country capacity. For comparisons, capacity data were standardized by dividing the total cold storage capacity by the population of children aged < 2 years in each country.

**Table 2:** Study assumptions.

	Routine immunization assumption	SARS-CoV-2 immunization assumption	Comments
**Vaccines**	WHO prequalified vaccines in multidose vial presentation given per WHO recommended schedule.^14^	SARS-CoV-2 vaccine in multidose vial presentation given as two doses, one month apart.	Given that there are no licensed SARS-CoV-2 vaccines, dose assumptions rely on dose schedule of SARS-CoV-2 vaccine clinical trials in the United States to date, and the volume analyses use packaging volumes per dose of AstraZeneca/Oxford University SARS-CoV-2 vaccine characteristics produced by Serum Institute of India.
**Packaging**	Tertiary packaging at the national level Secondary packaging at all subnational levels	Tertiary packaging at national level Secondary packaging at all subnational levels	National level vaccine volume analyses use total tertiary packaging volume required per dose (the unit for international transport), defined as the volume of the container holding cartons which contain vaccine vials divided by the total doses contained. Subnational levels vaccine volume analysis use total secondary packaging volume required per dose, defined as the volume of cartons which contain vaccine vials divided by the total doses contained.
**Storage temperature**	2° to 8 °C	2° to 8 °C	
**Coverage**	90% of target group	90% of target group	
**Target groups**	WHO recommended ages	Chronic diseases (any age) Persons ≥ 65 years Healthcare workers	Adults with chronic disease and older adults are at increased risk for severe SARS-CoV-2 disease [7]. Health care workers are at increased risk for SARS-CoV-2 infection and disease [7].
**Strategy**	Year round	Four month mass vaccination campaign	Once pandemic vaccines are available, there will be an imperative to deliver them expeditiously.
**Wastage multidose vials**	25%	3%	Vaccine wastage is the doses that are lost or unused. Routine immunization inputs are from WHO guidance [25]. SARS-CoV-2 wastage are from assumptions provided by Gavi to countries [13].
**Reserve stock**	3 months at national level 1 month at district and regional levels 0.5 months at health facility level	No reserve stock	Vaccine reserve stock are the excess supply in case of increased demand or stock-outs. Routine inputs are from WHO guidance while SARS-CoV-2 inputs assume high global demand and limited supply [12], [22], [26].
**Resupply intervals**	3 months at national, district, and regional levels 1 month at health facility level	Every month	Three month supply interval is common for routine immunization in low resource settings [24], while the SARS-CoV-2 supply interval assumes high global demand and limited supply.
**Vaccinators**	Nurse density per capita for WHO African Region countries multiplied by the simulated country population, the proportion of nurses providing immunization services, and estimates of absenteeism at baseline and during the SARS-CoV-2 pandemic	Same as for routine	Typically, persons delivering vaccines in the region are nurses, but not all nurses engage in the provision of immunization services. The estimates for nurses per capita are from WHO [18]. Estimates of percentage of nurses providing immunization services are from the Organisation for Economic Co-operation and Development [27]. Absenteeism estimates from observational data (baseline) and modelling data (SARS-CoV-2) from the United States [28], [29].

### 2.4 Analysis

We calculated the monthly doses and cold storage volumes required for routine immunization programs and SARS-CoV-2 vaccination campaigns to reach 90% of target populations [21]. We developed a vaccine flow-down schematic (Supplemental Figure 1) to depict the quantity of routine vaccine doses maintained at each immunization system level by month under normal circumstances and used it to calculate the total quantity of vaccine doses and their storage volumes at each level. To assess the feasibility of storing SARS-CoV-2 vaccines, we compared the anticipated vaccine volumes for routine immunization and SARS-CoV-2 vaccination campaigns to actual vaccine cold storage capacities at national and subnational levels under normal circumstances. We defined “immunization program workloads” as the doses delivered per vaccinator. To determine this, we calculated the number of monthly doses for each activity divided by the estimated number of vaccinators available in the country. We obtained estimates of the number of nurses for countries from a WHO Workforce Database [18], and we calculated nurse density per 10,000 population using 2017 country population estimates [14]. We determined the values for 25th percentile, 50th percentile, 75th percentile, and upper range nurse density for African Region countries, and we determined median values for other WHO Regions. To assess the range of immunization program workloads that would be experienced across the African Region, we applied the different nurse density estimates to the simulated country. We multiplied each nurse density estimate by 46%, the proportion of nurses estimated to provide immunization services [18], [27]. We assumed a 3% baseline absenteeism and an additional 8% absenteeism for SARS-CoV-2 pandemic months [28], [29]. To place results within the global context, we compared these results to calculations using the median number of vaccinators per capita from other WHO regions, while maintaining all other parameters unchanged. For the monthly comparisons and time series, we assumed constant routine immunization demand throughout the year and constant SARS-CoV-2 immunization demand during a mass vaccination campaign of four months duration. We performed all analyses using standard spreadsheet software including the made-for-purpose “WHO Vaccine Volume Calculator 2012” (Microsoft Corp, Redmond, WA, US) [30]. The WHO Vaccine Volume Calculator is designed to estimate the net storage volume of vaccines per child. Its specific purpose is to analyze how changes to the national immunization program schedule will affect cold chain capacity. As there was no involvement of human participants or personal identifiable information, institutional review board approval was not required.

## 3 Results

### 3.1 Description of population and vaccination target groups

We simulated a country of 20 million people in the African Region (Table 3). The percentage of the total population within each risk group was 3.1% for persons ≥ 65 years, 10.4% for persons with chronic diseases, 0.1% for HCWs, and 11.8% for all risk groups combined.

**Table 3:** Total and SARS-CoV-2 vaccine target group populations by age group.

**Age group**	**Total population**	**≥65 years**		**Chronic diseases**		**HCWs**		**All risk groups combined**	
		n	**% of total age group population**	n	**% of total age group population**	n	**% of total age group population**	n	**% of total age group population**
Under 5	3,139,586	0	0.0%	50,940	1.6%	0	0.0%	50,940	1.6%
5 to 9	2,828,076	0	0.0%	45,886	1.6%	0	0.0%	45,886	1.6%
10 to 14	2,500,943	0	0.0%	40,578	1.6%	0	0.0%	40,578	1.6%
15 to 19	2,128,524	0	0.0%	240,909	11.3%	0	0.0%	240,909	11.3%
20 to 24	1,817,105	0	0.0%	205,662	11.3%	2,842	0.2%	208,183	11.5%
25 to 29	1,559,398	0	0.0%	176,495	11.3%	2,842	0.2%	179,015	11.5%
30 to 34	1,331,654	0	0.0%	150,718	11.3%	2,842	0.2%	153,239	11.5%
35 to 39	1,109,669	0	0.0%	125,594	11.3%	2,842	0.3%	128,114	11.5%
40 to 44	898,313	0	0.0%	101,672	11.3%	2,842	0.3%	104,193	11.6%
45 to 49	717,631	0	0.0%	81,222	11.3%	2,842	0.4%	83,743	11.7%
50 to 54	571,040	0	0.0%	174,724	30.6%	2,842	0.5%	176,697	30.9%
55 to 59	448,210	0	0.0%	169,635	37.8%	2,842	0.6%	171,402	38.2%
60 to 64	338,046	0	0.0%	156,064	46.2%	2,842	0.8%	157,595	46.6%
65 to 69	238,867	238,867	100.0%	134,012	56.1%	0	0.0%	238,867	100.0%
70 + years	372,940	372,940	100.0%	225,615	60.5%	0	0.0%	372,940	100.0%
Total	20,000,000	611,807	3.1%	2,079,729	10.4%	25,580	0.1%	2,352,301	11.8%

Notes: 1. Total population of simulated country with the age distribution of the African Region per 2017 [14]. 2. From estimates of chronic diseases risk factors for severe SARS-CoV-2 illness for Africa [17]. 3. HCWs population used per capita estimates from the WHO African Region (12.79 per 10,000 population) multiplied by the simulated country population and distributed evenly from ages 20 to 64 [18]. 4. The sum of populations in the ≥ 65 years, chronic diseases, and HCW groups does not equal the population of all risk groups combined, as the HCW population was adjusted to account for persons with chronic diseases.

### 3.2 Vaccine doses

**Fig. 1:**
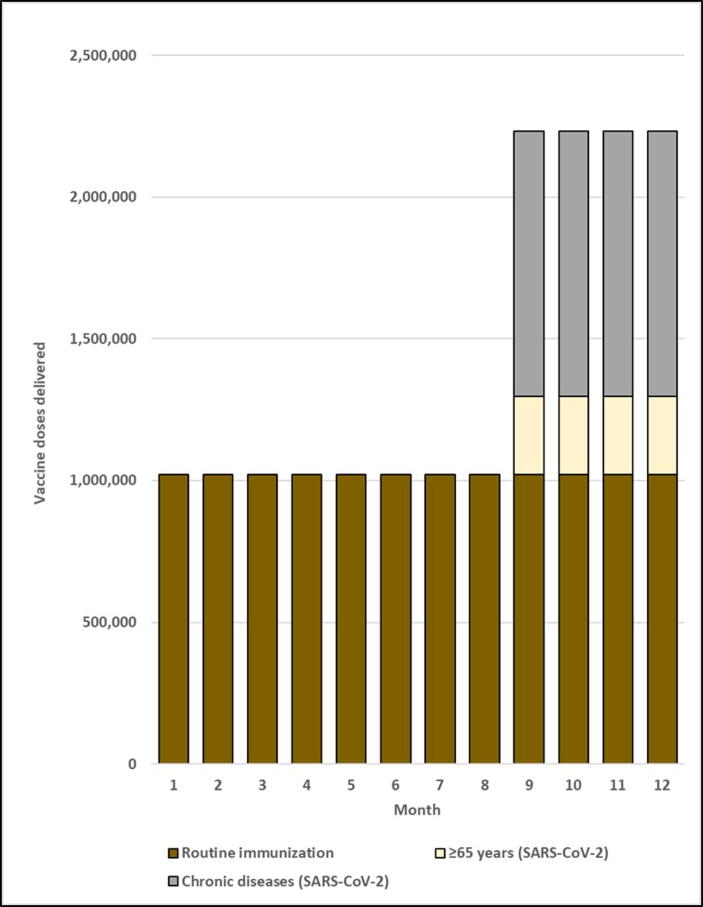
Monthly doses delivered for routine and SARS-CoV-2 risk group vaccination. Notes: 1. Figure includes sufficient doses for 90% coverage of target groups. 2. Figure assumes constant routine immunization demand over the year and constant SARS-CoV-2 immunization demand over a four-month mass vaccination campaign. 3. For illustrative purposes only, the mass vaccination campaign is conducted during months 9 through 12. 4. HCWs target group removed because volume contribution was too small to depict in the Figure.

As in most developing countries, all routine vaccination services in the simulated country would be received by children aged < 5 years and 9 through 14 years. Assuming systems would also be in place to provide immunization services to HCWs, established routine immunization platforms would reach 31.0% of the national population. A total of 1,020,931 routine vaccine doses would be given each month. Each month during the SARS-CoV-2 vaccination campaign, 275,313 doses would be delivered targeting persons ≥ 65 years, 935,878 targeting persons with chronic diseases, 11,511 targeting HCWs, and 1,058,536 targeting all risk groups combined (Table 4 and Fig. 1). Targeting SARS-CoV-2 vaccination of risk groups would increase monthly doses administered over the routine immunization baseline by 27.0% for persons ≥ 65 years, 91.7% for persons with chronic diseases, 1.1% for HCWs, and 103.7% for all risk groups combined.

**Table 4:** Monthly vaccine doses for routine immunization and SARS-CoV-2 mass vaccination campaigns by target group.

Age group	Routine immunization (doses)	≥65 years (SARS-CoV-2 vaccine doses)	Chronic diseases (SARS-CoV-2 vaccine doses)	HCWs (SARS-CoV-2 vaccine doses)	All risk groups combined (SARS-CoV-2 vaccine doses)
Under 5	939,468	0	22,923	0	22,923
5 to 9	81,462	0	20,649	0	20,649
10 to 14	0	0	18,260	0	18,260
15 to 19	0	0	108,409	0	108,409
20 to 24	0	0	92,548	1,279	93,682
25 to 29	0	0	79,423	1,279	80,557
30 to 34	0	0	67,823	1,279	68,958
35 to 39	0	0	56,517	1,279	57,651
40 to 44	0	0	45,753	1,279	46,887
45 to 49	0	0	36,550	1,279	37,684
50 to 54	0	0	78,626	1,279	79,514
55 to 59	0	0	76,336	1,279	77,131
60 to 64	0	0	70,229	1,279	70,918
65 to 69	0	107,490	60,305	0	107,490
70 + years	0	167,823	101,527	0	167,823
Total	1,020,931	275,313	935,878	11,511	1,058,536

Immunization program workload	Routine immunization (monthly doses / vaccinator)	≥65 years (monthly SARS-CoV-2 vaccine doses / vaccinator)	Chronic diseases (monthly SARS-CoV-2 vaccine doses / vaccinator)	HCWs (monthly SARS-CoV-2 vaccine doses / vaccinator)	All risk groups combined (monthly SARS-CoV-2 vaccine doses / vaccinator)

Routine immunization (prepandemic baseline)	165.3	–	–	–	–
Routine immunization (pandemic)	179.7	48.5	164.7	2.0	186.3
% of routine immunization prepandemic baseline	108.7%	29.3%	99.6%	1.2%	112.7%

Notes: 1. Analysis assumes 90% coverage of target groups and a two-dose series for SARS-CoV-2 vaccines 2. We assumed that both HPV doses were delivered to girls at 9 years of age. 3. Vaccinators = nurse density per capita (6.9 per 10,000) × country population (20,000,000) × percent of nurses that provide immunization services (46%). Nurse data are from WHO Global Health Workforce Statistics [18]. We used the median value from African Region countries at the most recent available date and divided by country population estimates to calculate per capita values [14]. 4. Adjustments account for 3% absenteeism (baseline) and an additional 8% absenteeism (during pandemic) [28], [29].

### 3.3 Immunization workload

Using the median value of nurse density from countries in the African Region (6.9 per 10,000 population), we estimated 13,840 nurses in the simulated country, of which an estimated 6,366 (46%) would provide vaccination services. Accounting for baseline and pandemic absenteeism, one vaccinator would administer 165.3 routine doses per month at baseline and 179.7 routine doses per month during the pandemic (Table 4).

**Fig. 2:**
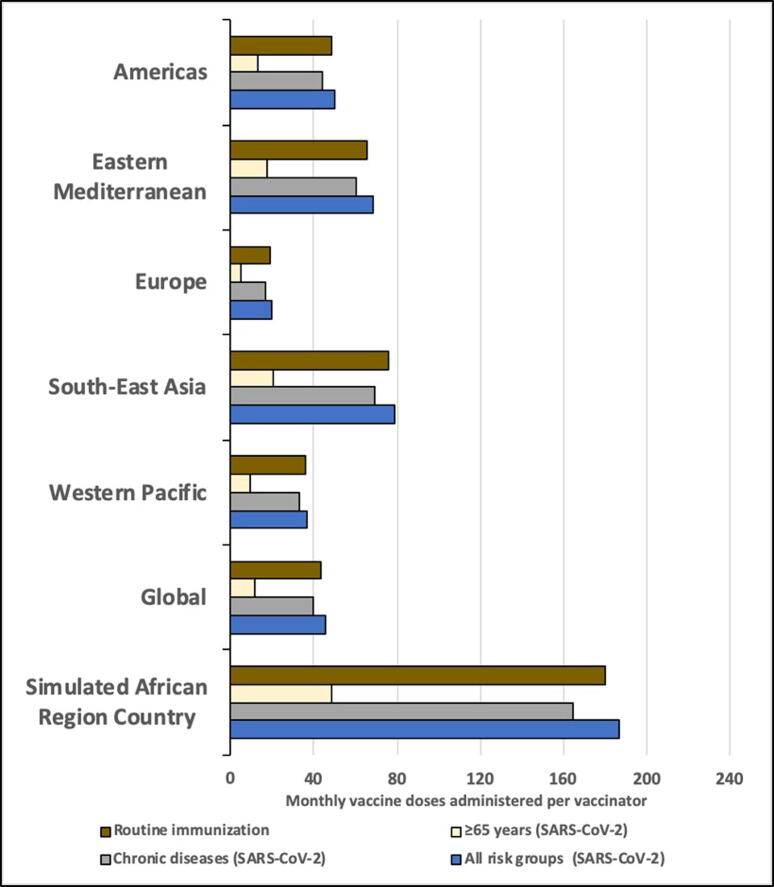
Monthly routine and SARS-CoV-2 campaign vaccine doses per vaccinator, by WHO Region estimated nurse density. Notes: 1. Data points represent the total monthly vaccines delivered divided by the total number of vaccinators in a country. Vaccinators = nurse density per capita × country population (20,000,000) × percent of nurses that provide immunization services (46%). 2. Nurse data are from WHO Global Health Workforce Statistics [18]. We used the median value from countries at the most recent available date and divided by country population estimates [14] to calculate per capita values and then categorized by WHO Region. 3. Routine immunization programs account for 3% absenteeism (baseline). SARS-CoV-2 vaccination programs account for 3% absenteeism (baseline) and an additional 8% absenteeism (during pandemic). 4. HCWs target group removed because volume contribution was too small to depict in the figure.

During the SARS-CoV-2 vaccination campaign, the number of persons vaccinated monthly per vaccinator would be 48.5 targeting persons ≥ 65 years, 164.7 targeting persons with chronic diseases, 2.0 targeting HCWs, and 186.3 targeting all risk groups combined. Compared to routine vaccines given at baseline, SARS-CoV-2 vaccination of risk groups would increase the immunization program workload for vaccinators by 29.3% for persons ≥ 65 years, 99.6% for persons with chronic diseases, 1.2% for HCWs, and 112.7% for all risk groups combined. Due to nursing personnel shortages in the African Region, the immunization program workload would be substantially lower if nurse density were similar to other WHO Regions. Repeating the analysis using nurse density estimates from other WHO Regions (but maintaining all other parameters unchanged), the immunization program workload would increase by 26.9% using estimates from the Americas, 36.6% using estimates from the Eastern Mediterranean, 10.4% using estimates from Europe, 42.2% using estimates from South-East Asia, 19.8% using estimates from the Western Pacific, and 24.3% using global estimates (with the relative proportion changes being the same regardless of risk group) (Supplemental Table 2 and Fig. 2).

We repeated the workload analysis using quartiles and upper range estimates for nurse density from countries in the African Region (while maintaining all other parameters unchanged). For each nurse density value, we estimated the following number of vaccinators: 25th percentile: 4,177; 50th percentile: 6,366; 75th percentile: 12,654; and upper range value: 62,957 (Supplemental Table 3 and Supplemental Figure 4). The 75th percentile nurse density estimate (13.8 per 10,000 population) was still lower than all other WHO Region nurse density estimates (with the closest being South-East Asia at 16.4 per 10,000 population). The upper range value (68.4 per 10,000 population) exceeded the nurse density estimate for all other WHO Regions (i.e. higher than the 66.5 per 10,000 population estimate for the European Region). Additional monthly doses per vaccinator for SARS-CoV-2 risk groups ranged from 4.9 to 73.9 targeting persons ≥ 65 years, 16.7–251.1 targeting persons with chronic diseases, 0.2–3.1 targeting HCWs, and 18.8–284.0 targeting all risk groups combined.

### 3.4 Routine vaccine storage

**Fig. 3:**
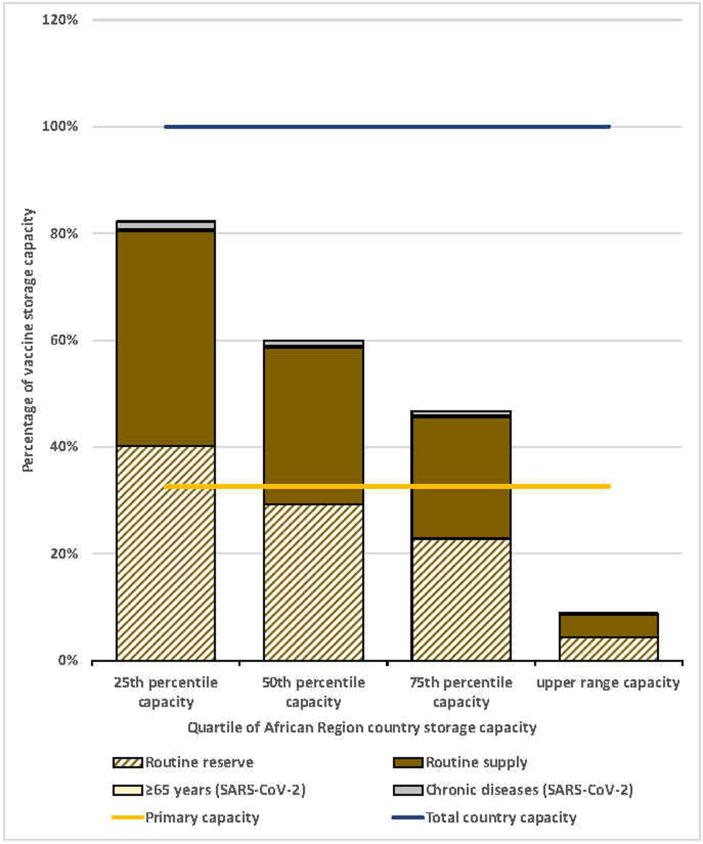
Maximum national level monthly routine and SARS-CoV-2 vaccine volumes as a proportion of total country capacity, by quartile of African Region country storage capacity. Notes: 1. Based on the maximum monthly national level vaccine storage volume (month 10) of the vaccine flow-down schematic (Fig. 1). 2. HCWs target group removed because volume contribution was too small to depict in the figure.

**Fig. 4:**
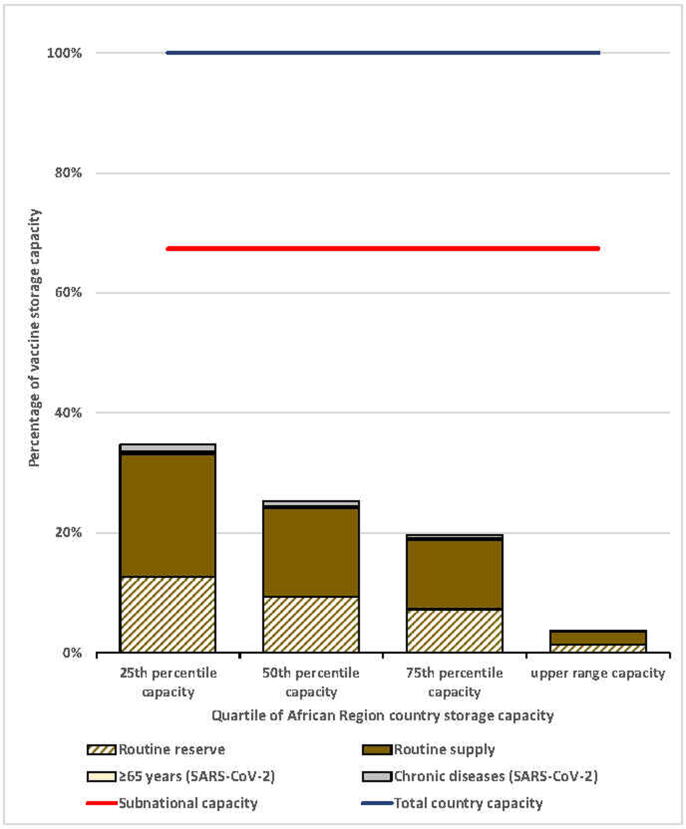
Maximum subnational level monthly routine and SARS-CoV-2 vaccine volumes as a proportion of total country capacity, by quartile of African Region country storage capacity. Notes: 1. Based on the maximum monthly subnational vaccine storage volume (month 11) of the vaccine flow-down schematic (Fig. 1). 2. HCWs target group removed because volume contribution was too small to depict in the figure.

We applied standardized African Region vaccine cold storage capacity range and quartiles to the simulated country. The total storage capacity would range from 38,403 L to 1,605,826 L, and national-level storage capacity would range from 12,535 L to 524,141 L (Table 5). We assessed the storage capacity during the month with the highest vaccine volumes. Using the vaccine flow-down schematic and adding the SARS-CoV-2 vaccination campaign during months 9 through 12, the maximum vaccine volumes were during month 10 for the national level and month 11 for the subnational levels (Supplemental Figures 2 and 3). We found that the maximum monthly routine vaccine volumes for the national level would be 139,161 L and for the aggregate subnational levels would be 57,472 L. At the national level, only the highest value capacity estimate had sufficient cold storage capacity to accommodate all routine vaccines according to our vaccine storage assumptions. Routine vaccine volumes exceeded that available at the national level for at least 75% of African Region country storage capacities (Fig. 3). At subnational levels, substantial excess available space existed for all capacities assessed, indicating that subnational levels had sufficient storage capacity for SARS-CoV-2 vaccines for at least 75% of countries (Fig. 4).

**Table 5:** M**aximum monthly storage volume for routine and SARS-CoV-2 vaccines by quartile of African Region country storage capacity.**

		Lower range capacity	25th percentile capacity	50th percentile capacity	75th percentile capacity	Upper range capacity
Country vaccine storage capacity	% of total country capacity	volume (L)	volume (L)	volume (L)	volume (L)	volume (L)
Total country capacity	100.0%	38,403	173,159	237,433	304,609	1,605,826
National level	32.6%	12,535	56,519	77,498	99,424	524,142
Subnational level	67.4%	25,868	116,640	159,935	205,185	1,081,685

**National level maximum monthly vaccine volumes**	Vaccine volume (L)	% of national level capacity	% of national level capacity	% of national level capacity	% of national level capacity	% of national level capacity

Routine immunization	139,161	1110.2%	246.2%	179.6%	140.0%	26.6%
≥65 years (SARS-CoV-2)	740	5.9%	1.3%	1.0%	0.7%	0.1%
Chronic diseases (SARS-CoV-2)	2,516	20.1%	4.5%	3.2%	2.5%	0.5%
HCWs (SARS-CoV-2)	31	0.2%	0.1%	<0.1%	<0.1%	<0.1%
All risk groups combined (SARS-CoV-2)	2,846	22.7%	5.0%	3.7%	2.9%	0.5%

**Subnational level maximum monthly vaccine volumes**	Vaccine volume (L)	% of subnational level capacity	% of subnational level capacity	% of subnational level capacity	% of subnational level capacity	% of subnational level capacity

Routine immunization	57,472	222.2%	49.3%	35.9%	28.0%	5.3%
≥65 years (SARS-CoV-2)	596	2.3%	0.5%	0.4%	0.3%	0.1%
Chronic diseases (SARS-CoV-2)	2,024	7.8%	1.7%	1.3%	1.0%	0.2%
HCWs (SARS-CoV-2)	25	0.1%	<0.1%	<0.1%	<0.1%	<0.1%
All risk groups combined (SARS-CoV-2)	2,290	8.9%	2.0%	1.4%	1.1%	0.2%

Notes: 1. We developed a vaccine flow-down schematic (Supplemental Fig. 1) to depict the routine vaccine doses maintained at each immunization system level by month and used it to calculate the total monthly vaccine doses and volumes stored throughout the immunization system. This table uses the maximum monthly national level (month 10) and subnational level (month 11) vaccine storage volumes from the schematic. 2. The vaccine storage capacity for Gavi-eligible African Region countries was standardized by dividing by the < 2 years country population in 2017. The quartiles and upper range values were calculated and then applied to the simulated African country. We used the median percentage of national level stores to total stores (32.6%) and health facility stores to total stores (34.9%) from the same data set for the analyses. 3. Immunization system assumptions are in Table 2.

### 3.5 SARS-CoV-2 vaccine storage

We estimated the volume required to store SARS-CoV-2 vaccines in tertiary packaging at the national level and in secondary packaging at subnational levels (Supplemental Figures 2 and 3). At the national level, monthly SARS-CoV-2 vaccines would occupy 740 L for persons ≥ 65 years, 2516 L for persons with chronic diseases, 31 L for HCWs, and 2,846 L for all risk groups combined (Table 5). Excluding the lowest range value for country capacity, the percentage of total national-level stores used by these vaccines by quartile of African Region country capacities ranged from 0.1% to 1.3% for persons ≥ 65 years, 0.5% to 4.5% for persons with chronic diseases, <0.1% to 0.1% for HCWs, and 0.5% to 5.0% for all risk groups combined (Table 5 and Fig. 3). At the subnational levels, monthly SARS-CoV-2 vaccines targeting risk groups would occupy 596 L for persons ≥ 65 years, 2,024 L for persons with chronic diseases, 25 L for HCWs, and 2,290 L for all risk groups combined. Excluding the lowest range value for country capacity, the percentage of subnational-level stores used by these vaccines by quartile of African Region country storage capacity ranged from 0.1% to 0.5% for persons ≥ 65 years, 0.2% to 1.7% for persons with chronic diseases, <0.1% to < 0.1% for HCWs, and 0.2% to 2.0% for all risk groups combined (Table 5 and Fig. 4).

## 4 Discussion

We used WHO tools and guidelines to estimate the effect of deploying SARS-Cov-2 vaccines on cold storage capacity and immunization workload in the African Region [19], [24]. This analysis does not take the place of country-level assessments using national contexts and data for deployment planning, but it does describe the potential impact of SARS-CoV-2 mass vaccination campaigns may have on country immunization systems in the region. While we simulated a country of 20 million population for this analysis, we used actual immunization, population, healthcare worker density, cold storage capacity data available for the region, and approved SARS-CoV-2 vaccine characteristics. Notably, we assumed the SARS-CoV-2 vaccines required a two-dose series.

Our analysis revealed that the national-level storage capacity would be insufficient to accommodate additional SARS-CoV-2 vaccines for a vaccination campaign targeting risk groups as large as persons ≥ 65 years (3.1% of the population) in at least 75% of African Region countries. However, our analysis also showed that all African Region countries likely have sufficient subnational-levels storage capacity to accommodate SARS-CoV-2 vaccines for mass vaccination campaigns, though much care and planning may be necessary to ensure adequate surge capacity. This means that there is insufficient excess cold storage capacity at the national level, whereas once vaccines are distributed to subnational levels in the health system there should be adequate storage capacity. This bottleneck in the vaccine cold chain at the national level can be addressed in advance by decreasing reserve stocks, shortening supply intervals, and removing diluents from cold chain when possible, removing products from tertiary packaging earlier in the chain, distributing vaccines to the subnational levels, and/or installing or leasing more refrigeration capacity. However, implementation of SARS-CoV-2 vaccination campaigns will also require increases in accompanying dry goods, such as syringes, safety boxes, cotton, alcohol, personal protective equipment, and other essential pieces of equipment necessitating additional planning and budget for operations and waste management. More in-country vaccine shipments will be required to distribute routine and SARS-CoV-2 vaccines if transportation capacities are not increased, adding costs and logistical challenges. Care should be taken not to disrupt routine vaccine supply and distribution, as stockouts of routine vaccines will exacerbate national immunization programs already impacted by the pandemic. Further, large quantities of unused and unexpired vaccines due to disruptions in the routine vaccination can affect available storage capacity for SARS-CoV-2 vaccines.

While we found that the aggregate subnational-level storage capacities are adequate for SARS-CoV-2 vaccines, with very large margins expected for a majority of countries, it should be noted that it is not possible nor practical to fill all available subnational-level storage capacity. For example, at the health facility level, standard vaccine refrigerators may have multiple times the capacity needed for vaccines used at that facility, but that excess capacity cannot be easily used to store vaccines for other facilities. The complicated logistics, including planning, implementation, and coordination of operations, required to fully utilize the available storage capacity at the lower levels of the cold chain makes it impractical. Nevertheless, our findings show that there is ample vaccine storage capacity available at subnational levels, and creative management of both routine and campaign vaccines could leverage some of that excess capacity during the SARS-CoV-2 vaccination campaign period.

Another major obstacle for SARS-CoV-2 vaccine delivery in the African Region will be the availability of qualified vaccinators. The African Region has the smallest health workforce of any WHO Region [18], and the workload required to vaccinate risk groups would increase by 27.0% for persons ≥ 65 years and 91.7% for persons with chronic diseases, compared to routine immunization baselines. SARS-CoV-2 vaccination activities targeting HCWs will require less additional workload given their small population size and ease of access to vaccination services. We did not account for immunization system personnel who do not provide immunization services, but they will be critical for coordination, operations, and management of supply chain. Further, staff will be called upon to do many tasks outside their traditional duties. Our analysis highlights the need for significant planning, and investment in equipment, training, and logistics for SARS-CoV-2 vaccine deployment.

There is reason for optimism that the African Region SARS-CoV-2 vaccination response will be much improved from the 2009 H1N1 pandemic experience. Over the last ten years, a concerted global effort has greatly improved cold chain infrastructures and increased storage capacity [9], [10]. Furthermore, countries in the African Region have successfully implemented mass vaccination campaigns with several different vaccines. From 2010 to 2017, the MenAfriVac introduction strategy targeted all persons aged one through 29 years in 23 Meningitis Belt countries [31]. From 2010 to 2011, six West African countries implemented mass vaccination campaigns achieving 98% coverage in the target population [31]. Over a ten-day period in Burkina Faso, a team of 5,328 vaccinators and as many volunteers vaccinated 11,425,391 persons (73% of the country population) [31]. The lyophilized vaccine was supplied in 10-dose vials, requiring reconstitution [19]. Each vaccinator vaccinated an average of 214 persons daily. The Region has had many other successful vaccination campaigns in the last decade, as well, including with vaccination against polio, measles, and yellow fever [32], [33], [34], [35]. Given likely SARS-CoV-2 vaccine supply limitations [7], the impact on cold storage and workforce may be lessened, as WHO is currently planning for an initial tranche of vaccines to cover 3% of a country’s population [36]. With sufficient planning and resources, success could be achieved for SARS-CoV-2 vaccination programs targeting much higher proportion of a country’s population.

A SARS-CoV-2 vaccination campaign would be greatly facilitated if vaccine delivery were simplified [23]. While a one dose vaccine would be ideal, products achieving WHO Emergency Use Listing as of February 2021 all require two-dose schedules[2]. Nasal spray, transdermal patch, or oral vaccines that could be administered by trained lay persons would free skilled health workers to provide other essential services, but no such vaccines are in advanced human trials as of February 2021 [1]. While single dose vial vaccine presentations limit vaccine wastage [4], [22], and prefilled syringe presentations limit wastage and greatly simplify administration [22], their cold storage volumes per dose make these presentations highly problematic for use in mass vaccination campaigns in the African Region.

WHO has advised that certain products would be challenging to include in national immunization schedules if they do not meet specific Programmatic Suitability Criteria [37]. Vaccine products must meet these mandatory criteria to achieve WHO prequalification: include an anti-microbial preservative (for injectable liquid vaccines in multi-dose containers), be relatively thermostable (not requiring storage at less than −20 °C), have a dose volume ≤ 1 ml (for injectable vaccines), and not require intravenous administration [37]. Exceptions to the programmatic suitability criteria are made for the prequalification of certain emergency-use vaccines, such as for the prevention of Ebola Virus Disease, which are stored at −80 °C to −60 °C [31]. The first SARS-CoV-2 vaccine candidates to enter human trials in the United States used a mRNA platform [1], [2]. Most mRNA vaccines are frozen for storage requiring special storage, transport, and handling conditions that are not required for all routine vaccines. DNA vaccine candidates are more thermostable, but would likely require administration via electroporation devices. Any licensed products requiring freezing for storage or nonstandard devices for vaccine administration would be prohibitive to use at scale in developing countries.

Our study should be interpreted in light of its strengths and limitations. Our use of real-world data inputs and WHO tools and guidance strengthen the generalizability of the results and comparability to other cold chain capacity assessments. If a SARS-CoV-2 vaccine requiring only one dose for protection is eventually licensed, the estimated doses and storage volumes required would substantially decrease. Conversely, if only products requiring ultra-low temperature cold chain or extra effort and training to administer are licensed, the operational feasibility of mass vaccination campaigns for developing countries would be very limited. We applied WHO recommended routine vaccination schedules and product characteristics [19], [38], however, as of 2017, some of the routine vaccines we assumed for the simulated country had limited adoption in African Region countries (Supplemental Table 1). If we overestimated routine vaccine doses and volumes, then there would be even more available capacity for SARS-CoV-2 vaccines. We used estimates of percentage of nurses providing immunization services from OECD countries [27], but the proportion of doses per vaccinator for SARS-CoV-2 target groups divided by doses per vaccinator for routine immunization is unaffected by the estimate used. Not all cold storage space can be effectively utilized; we used total cold storage volumes and not effective cold storage volumes which is estimated to be two-thirds of total volumes [39]. Our analysis assumes 90% coverage; however, the operational needs will be decreased if there is <90% demand for SARS-CoV-2 vaccines among risk groups or if target coverage is lower. It is critical that pandemic vaccine response have health communications and social mobilization plans in place, and that efforts to understand and overcome vaccine hesitancy be made. While we used WHO-recommended chronic diseases prevalence estimates as risk factors for SARS-CoV-2 [16], some of these diseases may go underdiagnosed in the African Region, resulting in overestimates of vaccine demand for this risk group. We received vaccine cold storage capacity for Gavi-eligible countries. As Gavi has led the effort to strengthen cold storage capacities among countries receiving Gavi support, we do not know whether non-Gavi-eligible countries have comparable capacities to the Gavi-eligible countries which have received this support. Despite Gavi cold storage capacity investments, the WHO Immunization Practices and Advisory Committee (IPAC) noted in 2017 that there were weaknesses in developing country supply chain management which needed further investments and strengthening [40].

The development of candidate SARS-CoV-2 vaccines must take into account operational realities and programmatic suitability in developing countries. While we believe that African Region countries currently have sufficient overall cold chain capacity to accommodate SARS-CoV-2 mass vaccination campaigns, our study highlights the limitations in cold storage capacity at national levels and in workforce availability to administer these vaccines. Vaccination of risk groups with SARS-CoV-2 vaccines is possible with sufficient planning and infrastructure strengthening, but we anticipate risks to routine immunization and significant logistical challenges. Further, the development of products that are not programmatically suitable for use in developing country contexts will result in massive inequities, and concerted global efforts are urgently required to ensure that appropriate preventive interventions are made available to all who need them globally. Resources must be mobilized urgently to ensure that infrastructures and training are in place to maximize the impact of future SARS-CoV-2 vaccines in the African Region and other developing country settings.

## Declaration of Competing Interest

The authors declare that they have no known competing financial interests or personal relationships that could have appeared to influence the work reported in this paper.
